# Supplementary material for: Wave-particle energy exchange directly observed in a kinetic Alfvén-branch wave
Source: Nat Commun. 2017 Mar 31;8:14719. doi: 10.1038/ncomms14719 (PMC5380972; doi:10.1038/ncomms14719)
Supplement: Supplementary Information — Supplementary Figures. [file ncomms14719-s1.pdf]

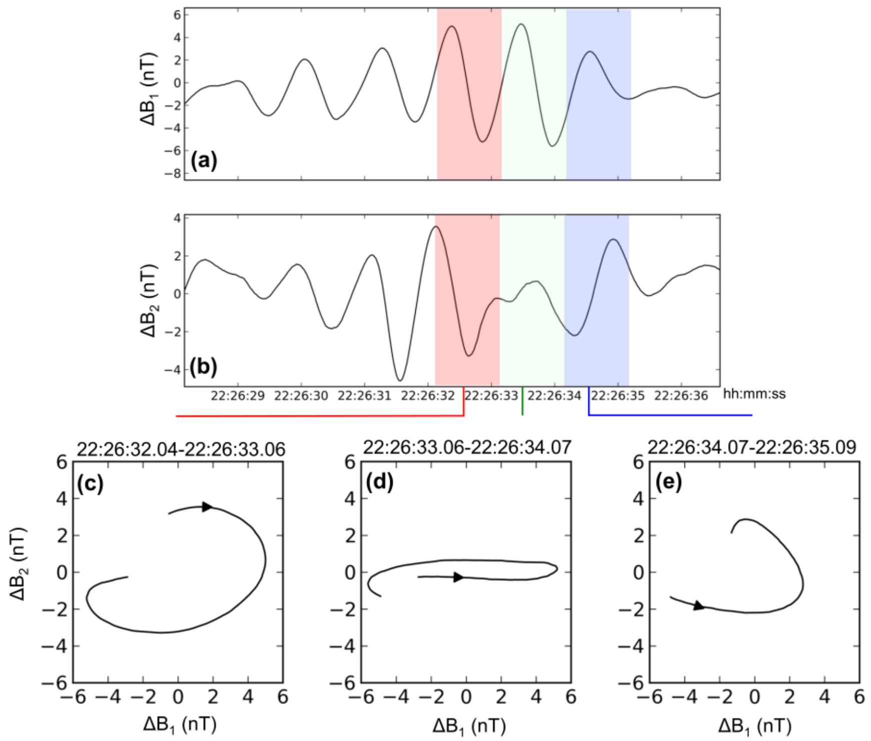

Supplementary Figure 1. Perpendicular magnetic field fluctuations. Transverse fluctuations of the MMS-averaged magnetic field components ΔB_1 and ΔB_2 are shown for a KAW packet in magnetic coordinates. Hodograms of ΔB_2 vs. ΔB_1 for three subintervals of the wave packet indicated a transition from left-handed to right-handed polarization near the center of the packet.

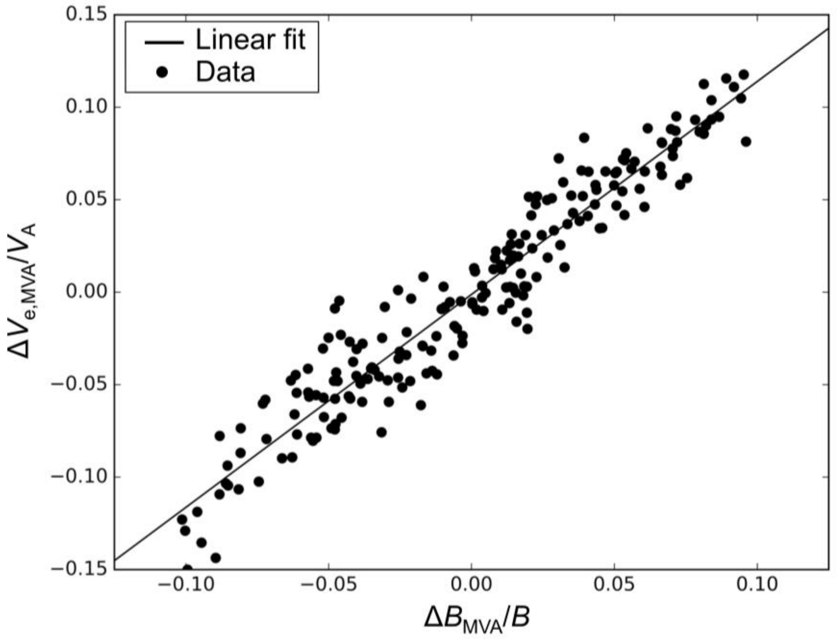

Supplementary Figure 2. Correlation of velocity and magnetic field fluctuations. $\Delta\mathbf{V}_e/V_A$ vs. $\Delta\mathbf{B}/B$ for transverse fluctuations in the direction corresponding to the minimum variance (MVA) direction of $\Delta\mathbf{J}$, i.e., [0.93,0.32,0.18]. The positive correlation between $\Delta\mathbf{V}_e$ and $\Delta\mathbf{B}$ indicated that $k_{||} < 0$, i.e., the wave propagated anti-parallel to the local magnetic field. The slope of a linear fit of the fluctuations was near unity (1.15±0.03), indicating that the parallel (with respect to the local magnetic field) wave speed was close to V_A. B = 55 nT and V_A = 380 km s^{-1} were used for the normalizations of magnetic and velocity fluctuations, respectively.

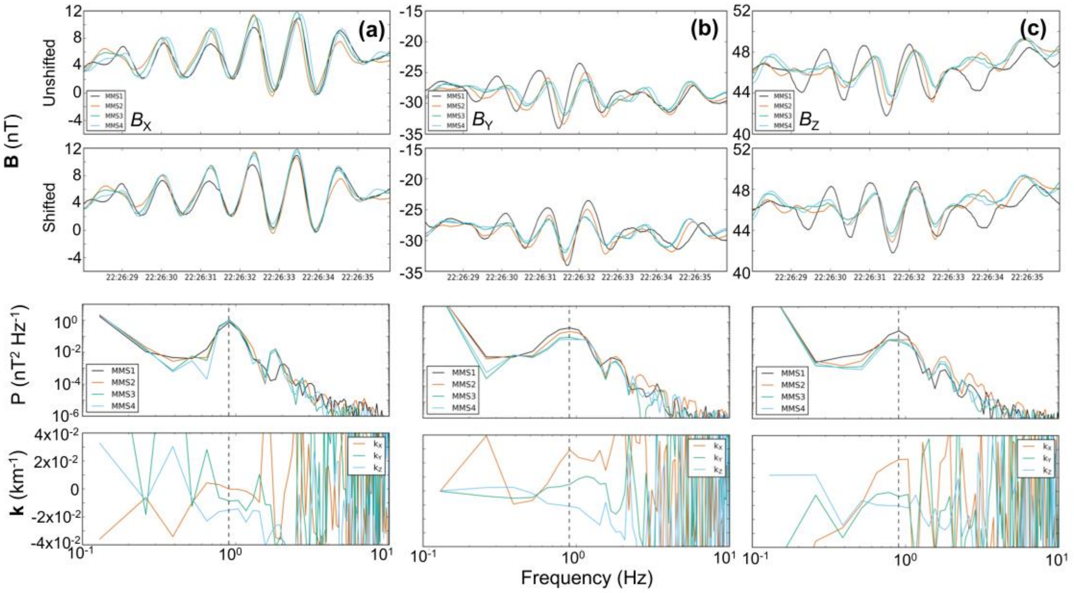

Supplementary Figure 3. Phase-differencing of magnetic field fluctuations. Measured and time-shifted magnetic field, power spectral density, and **k**-vector determined from phase differencing of (a) B_X, (b) B_Y, and (c) B_Z using MMS3 as a reference spacecraft. The phase difference at spectral density peak of ~0.9 Hz was used to shift the data. Different shifts of MMS1 data for different components of the magnetic field vector indicate that this wave packet is not truly planar. Nonetheless, the **k** vectors derived from each indicated $k_\perp \rho_i \sim 1$.
